# Dual antibody-aided mesoporous nanoreactor for H_2_O_2_ self-supplying chemodynamic therapy and checkpoint blockade immunotherapy in triple-negative breast cancer

**DOI:** 10.1186/s12951-023-02154-0

**Published:** 2023-10-24

**Authors:** Ying-Tzu Chen, Ying-Xiang Luo, Shih-Hsuan Chan, Wen-Yi Chiu, Hung-Wei Yang

**Affiliations:** 1 Department of Biomedical Engineering, National Cheng Kung University, Tainan, 70101 Taiwan; 2 Department of Neurosurgery, Neuroscience Research Center, Chang Gung Memorial Hospital, Linkou, 33305 Taoyuan Taiwan; 3 Institute of Medical Science and Technology, National Sun Yat-sen University, Kaohsiung, 80424 Taiwan; 4 School of Chinese Medicine, College of Chinese Medicine, China Medical University, Taichung, 40402 Taiwan; 5 Cancer Biology and Precision Therapeutics Center, China Medical University, Taichung, 40402 Taiwan; 6 Chinese Medicine Research Center, China Medical University, Taichung, 40402 Taiwan; 7 Department of Family Medicine, Kaohsiung Armed Forces General Hospital, Kaohsiung, 80284 Taiwan; 8 Medical Device Innovation Center, National Cheng Kung University, Tainan, 70101 Taiwan

**Keywords:** Triple-negative Breast cancer (TNBC), Mesoporous nanoreactors, Chemodynamic therapy (CDT), Checkpoint blockade immunotherapy, Collaborative treatment

## Abstract

Triple-negative breast cancer (TNBC) represents a formidable challenge due to the absence of estrogen receptor (ER), progesterone receptor (PR), and human epidermal growth factor receptor 2 (HER2) expression, rendering it unresponsive to conventional hormonal and targeted therapies. This study introduces the development of mesoporous nanoreactors (NRs), specifically mPDA@CuO_2_ NRs, as acid-triggered agents capable of self-supplying H_2_O_2_ for chemodynamic therapy (CDT). To enhance therapeutic efficacy, these NRs were further modified with immune checkpoint antagonists, specifically anti-PD-L1 and anti-CD24 antibodies, resulting in the formation of dual antibody-aided mesoporous nanoreactors (dAb_PD−L1/CD24_-mPDA@CuO_2_ NRs). These NRs were designed to combine CDT and checkpoint blockade immunotherapy (CBIT) for precise targeting of 4T1 TNBC cells. Remarkably, dAb_PD−L1/CD24_-mPDA@CuO_2_ NRs exhibited tumor-targeted CDT triggered by H_2_O_2_ and successfully activated immune cells including T cells and macrophages. This integrated approach led to a remarkable inhibition of tumor growth by leveraging the collaborative effects of the therapies. The findings of this study introduce a novel and promising strategy for the integrative and collaborative treatment of refractory cancers, providing valuable insights into addressing the challenges posed by aggressive breast cancer, particularly TNBC.

**Supplementary Information:**

The online version contains supplementary material available at 10.1186/s12951-023-02154-0.

## Introduction

Triple-negative breast cancer (TNBC) is a breast cancer subtype characterized by the absence of estrogen receptor (ER), progesterone receptor (PR), and human epidermal growth factor receptor 2 (HER-2) expression. TNBC is known for its high invasiveness, metastatic potential, propensity for relapse, and poor prognosis [1–3]. Due to the lack of ER, PR, and HER-2 receptors, hormone therapy and targeted therapies commonly used in clinical practice are ineffective, leaving patients with limited treatment options. Chemotherapy is the primary treatment modality; however, resistance to conventional therapies arises due to the overexpression of epidermal growth factor receptor (EGFR) proteins on the cell surface, leading to short-lived responses, severe side effects, and systemic toxicity [4, 5]. Additionally, monotherapy targeting TNBC-specific receptors has shown limited efficacy. For instance, although EGFR is highly expressed in 70–78% of basal-like TNBC cells [6], EGFR-targeted therapy alone has been unsatisfactory. Enhanced inhibitory effects necessitate the combined use of downstream signaling inhibitors [7]. Therefore, selecting appropriate specific receptors for TNBC is crucial, and standardized treatment approaches for TNBC remain elusive.

Considering these challenges, researchers turned to alternative approaches, such as immunotherapy, which showed promise in the treatment of TNBC. Immunotherapy involves harnessing the immune system’s functionality and specificity to treat malignant tumors. Tumor cells interact with T cells through antigen-presenting cells, and these interactions were facilitated by specific surface protein receptors that could either stimulate or inhibit T cell activity. Proteins that inhibited T cell activity were called tumor immune escape proteins or immune checkpoints [8, 9]. In recent years, many studies had pointed out that TNBC was more suitable for immunotherapy using immune checkpoint inhibitors (ICIs) compared to other subtypes of breast cancer. It was because TNBC had higher levels of tumor-infiltrating lymphocytes (TILs), higher PD-L1 expression on the tumor, and a greater number of nonsynonymous mutations. These characteristics provided direct targets for ICIs, correlated with better responses to ICIs in other tumors, and gave rise to tumor-specific neoantigens, which activated neoantigen-specific T cells to mount an antitumor immune response [10]. Not only was PD-L1 found to be highly expressed in TNBC, but CD24 and CD47 were also discovered to have a similar situation. However, unlike PD-L1, CD24 and CD47 protected cancer cells from attack by directly interacting with the Siglec-10 signaling pathway in macrophages. Therefore, it was necessary to block the connection between them using ICIs - CD24 and CD47 antibodies, enabling macrophages to begin phagocytosing cancer cells more effectively. In contrast to the anti-CD47 antibody, the anti-CD24 antibody demonstrated no detectable binding to human red blood cells, thereby significantly reducing toxicity. This is because CD47 is recognized as a transmembrane protein of human red blood cells [11, 12]. Therefore, CD24 is a potent and more appropriate anti-phagocytic “don’t eat me” signaling molecule that directly protects cancer cells from attack by Siglec-10-expressing macrophages. Previous research also confirmed that by downregulating the CD24 and CD47 proteins on breast cancer cells using the tumor suppressor gene ZBTB28, the phagocytic activity of macrophages increased. Effectively blocking both proteins inhibited the proliferation of late-stage breast cancer cells [13]. Therefore, in addition to PD-L1 inhibitors, CD24 inhibitors could have potentially emerged as a novel immunotherapeutic approach for treating TNBC. In the past, studies on melanoma treatment demonstrated that combination therapy had a higher objective response rate compared to monotherapy [14–16]. Consequently, in the previous TNBC treatment strategies, there was hope to enhance the anti-tumor immunotherapeutic effect through dual or triple blockade of immune checkpoints.

Although TNBC had a relatively higher response rate to ICIs, for many patients, the efficacy of monotherapy was still insufficient. As cancer treatment evolved, the current trend gradually shifted from a single treatment modality to combination therapies, aiming to multiply the therapeutic effects. In both early TNBC and metastatic TNBC, the combination of ICIs and chemotherapy demonstrated exceptional therapeutic effects, but it also led to an increase in side effects. These side effects included anemia, nausea, hair loss, fatigue, peripheral neuropathy, neutropenia, and hypothyroidism. These side effects might have had a certain impact on the patient’s quality of life and the smooth progress of treatment [17, 18]. Chemotherapy lacked tumor specificity, harming both tumor and normal cells, leading to side effects. Even with imaging-guided positioning, physical treatments like photothermal, ultrasound or radiation therapy might have caused damage to surrounding tissues or induced cancer metastasis [19]. Chemodynamic therapy (CDT) utilized the tumor microenvironment to destroy in situ tumors by delivering biocompatible catalysts that converted H_2_O_2_ into therapeutically effective reactive oxygen species (ROS) [20]. In comparison to photodynamic therapy, CDT did not rely on light, photosensitizers, or oxygen, reducing limiting factors [21]. Furthermore, its process of generating free radicals did not require oxygen, reducing dependency on other conditions. This treatment approach could reduce side effects experienced by patients during therapy and improve the challenges faced by other treatments in terms of tissue depth and hypoxic tumor microenvironments [22]. As a result, CDT was considered a promising novel cancer treatment strategy.

Nevertheless, owing to the intricate intracellular environment of tumor cells, the therapeutic efficacy of CDT is significantly limited. Integrating CDT with other treatment modalities has emerged as a burgeoning trend in cancer therapy. For example, the integration of CDT with photothermal therapy (CDT/PTT) and CDT with chemotherapy (CDT/chemotherapy) has demonstrated a significant increase in antitumor activity compared to individual treatments [23–25]. CDT relies on the generation of reactive oxygen species (ROS) to induce toxicity in cancer cells. A Fenton-like reaction is employed, wherein metal ions react with higher concentrations of H_2_O_2_ in the acidic tumor microenvironment, leading to the production of toxic ·OH [26]. Previously, numerous nanomaterials have utilized transition metals with catalytic activity to trigger chemical reactions of endogenous H_2_O_2_ within tumors, thereby generating free radicals that inhibit tumor formation. These transition metals, including Fe, Cu, Mn, and Co, have all been proven effective in inducing CDT [18]. Fe^2+^-mediated Fenton reactions require relatively high acidity, resulting in lower catalytic efficiency. In contrast, Cu^2+^-catalyzed Fenton-like reactions can increase the reaction rate by approximately 60 times compared to Fe^2+^ [27]. Despite the high efficiency of Cu^2+^-catalyzed Fenton-like reactions in weakly acidic and neutral media, Cu^2+^ easily dissolves in water, which may cause premature decomposition before reaching the tumor region. This could reduce the nanoparticle concentration in the tumor area and increase the risk to normal tissues. Consequently, an appropriate nanoparticle delivery platform is needed to encapsulate copper oxide nanoparticles, ensuring that the CDT reaction occurs exclusively within the tumor region. Thus, in addition to CDT, this study proposes the integration of immunotherapy as an alternative treatment approach to enhance the effectiveness of TNBC treatment while minimizing the impact on normal tissues and skin.

**Scheme 1:**
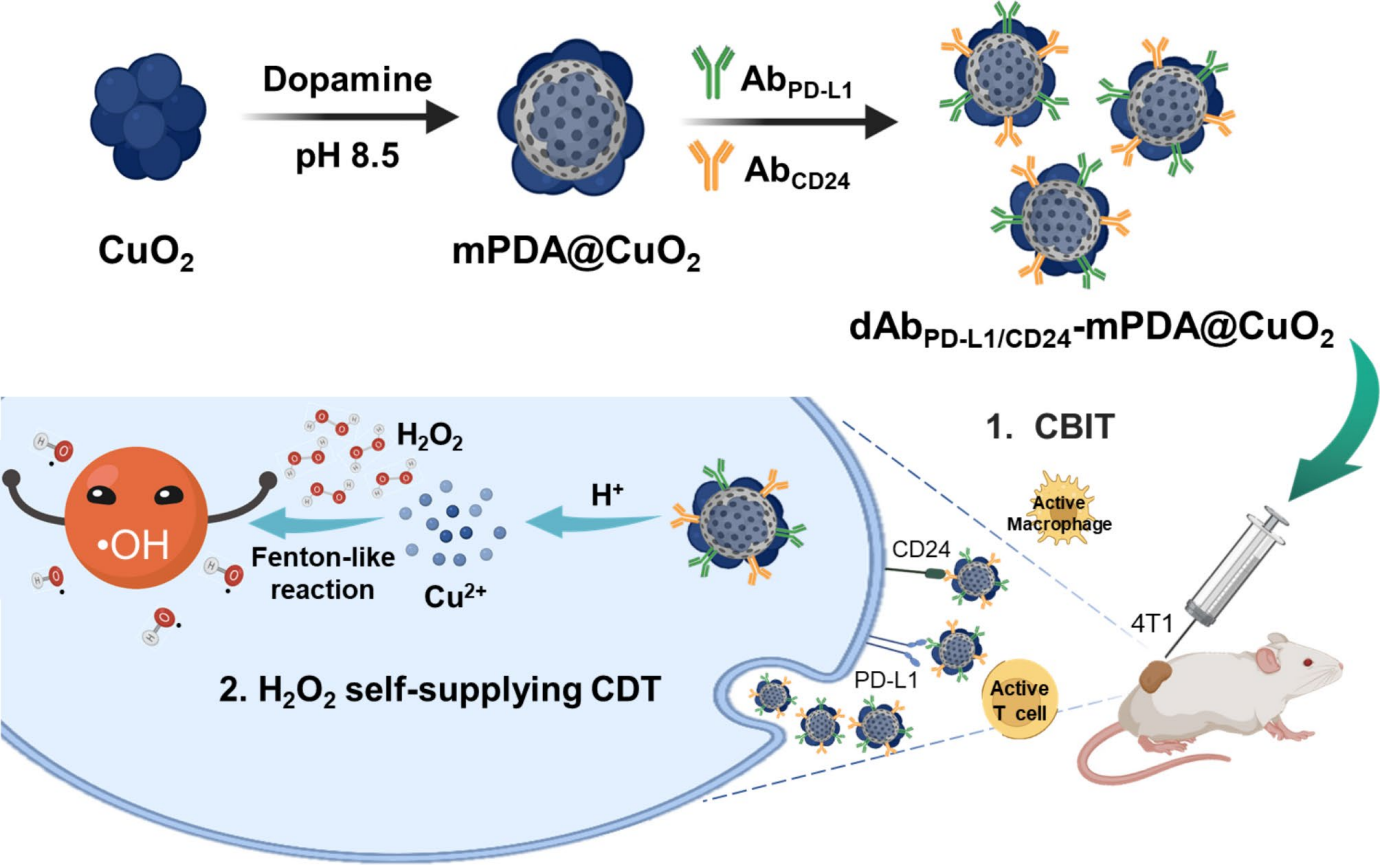
**(A)** Schematic illumination of the formation of dAb_PD−L1/CD24_-mPDA@CuO_2_ NRs as a nanotherapeutic agent for H_2_O_2_ self-supplying CDT and CBIT simultaneous in TNBC

In this study, the development of mesoporous nanoreactors (NRs) is reported, specifically mPDA@CuO_2_ NRs composed primarily of CuO_2_ and mPDA. The adhesive properties of the mPDA surface, inspired by mussels [28], were utilized to successfully conjugate antibodies onto the surface of mPDA@CuO_2_ NRs, resulting in the formation of dAb_PD−L1/CD24_-mPDA@CuO_2_ NRs (Scheme 1 A). These dual antibody-conjugated nanoreactors demonstrate potential for checkpoint blockade immunotherapy (CBIT) by effectively targeting and blocking PD-L1 and CD24 proteins present on breast cancer cells, specifically TNBC cells. Moreover, the dAb_PD−L1/CD24_-mPDA@CuO_2_ NRs exhibit targeted chemodynamic therapy (CDT) with the ability to self-supply H_2_O_2_ within the tumor microenvironment, leading to efficient suppression of 4T1 breast tumors (Scheme 1B). The findings highlight that the designed dAb_PD−L1/CD24_-mPDA@CuO_2_ NRs significantly enhance antitumor efficacy through the synergistic effects of H_2_O_2_ self-supplying CDT and CBIT, offering a promising therapeutic approach for breast cancer treatment, particularly for TNBC.

## Materials and methods

### Materials

Copper(II) chloride (CuCl_2_), hydrogen peroxide (H_2_O_2_, ~ 30%), Tris base, Pluronic® 127, dopamine, 3,3’,5,5’-tetramethylbenzidine (TMB), and bovine serum albumin (BSA) were purchased from Sigma-Aldrich (St. Louis, MO, USA). Ammonium hydroxide (NH_4_OH), ethanol (~ 99%), acetone (~ 99%), and mesitylene were purchased from J.T. Baker® (Pennsylvania, USA) and Alfa Aesar (Heysham, Lancashire) respectively. Cell culture-related products, including Dulbecco’s Modified Eagle Medium (DMEM, CAT: CC103-0500), RPMI 1640 medium (CAT: CC110-0500), and agarose, were obtained from GeneDireX, Inc. Penicillin-streptomycin (10,000 U/mL, CAT: 154140-122) and fetal bovine serum (FBS, CAT: 10437-028) were procured from Gibco®. Interleukin-2 (IL-2, CAT: 50,792-M08H) was procured from Sino Biological.

### Analysis of immune checkpoint protein expression

MDA-MB-468 (human), MDA-MB-231 (human), and 4T1 (mouse) breast cancer cells (3 × 10^5^ per well) in 6-well plates were incubated for 24 h and then washed three times with 2% FBS contained PBS (pH = 7.4). Followed by incubation with PD-L1 antibody (0.2 µg/1 × 10^6^ cells, ABflo®488 Rabbit anti-Human PD-L1/CD274 mAb, CAT: A22304, ABclonal; 5 uL/1 × 10^6^ cells, Rabbit anti-Mouse PD/L1 mAb-PE/Cy5.5 conjugated, CAT: MBS2558960 MyBioSourec) or CD24 antibody (0.5 µg/1 × 10^6^ cells, CD24 Rat mAb, FITC-conjugated, from eBioscience™, CAT: 12-5982-82, Invitrogen) for 1.5 h. Afterward, all samples were washed three times with PBS (pH = 7.4) enriched with 2% FBS, fixed with 1% paraformaldehyde, and finally quantified using the Attune Nxt flow cytometer (Thermo Fisher Scientific, USA).

### Preparation of dual antibody-aided mesoporous nanoreactors

#### Preparation of CuO_2_

Initially, 0.2 g of CuCl_2_ powder was dissolved in 5 mL of deionized water (DI-H_2_O). Subsequently, 0.16 mL of a 2.573 M H_2_O_2_ solution and 5 mL of a 0.05 M Tris solution at pH 8.5 were added to the CuCl_2_ solution. The mixture was thoroughly mixed at room temperature for 10 min. To remove excess reagents, the resulting CuO_2_ NRs were washed three times with ethanol through centrifugation at 12,000 rpm. Finally, the obtained CuO_2_ NRs were resuspended in DI-H_2_O for subsequent experiments.

#### Preparation of mPAD@CuO_2_

In brief, 180 mg of F127 powder was dissolved in 4.5 mL of ethanol and mixed thoroughly at room temperature until the solution transitioned from turbid to transparent. Subsequently, 4.5 mL of DI-H_2_O, 45 µL of mesitylene solution, and 18 mg of DA were added to the F127 solution. The resulting mixture was continuously stirred at room temperature for 10 min. To form PDA@CuO2, 180 µL of NH_4_OH was added to the solution mentioned above, and the mixture was stirred in the dark for 45 min. Unreacted substances in the supernatant were eliminated through centrifugation at 12,000 rpm. For the production of mesoporous PDA@CuO_2_ NRs (mPDA@CuO_2_ NRs), the PDA@CuO_2_ NRs were dispersed in a 1:1 (v:v) mixture of ethanol and acetone. The suspension was shaken for 30 min to remove the F127 template, followed by two washes with ethanol through centrifugation at 12,000 rpm (this etching process was repeated twice). Finally, the resulting precipitate was stored at 4 °C for subsequent experiments.

#### Preparation of Ab_PD−L1_-mPDA@CuO_2_ NRs, Ab_CD24_-mPDA@CuO_2_ NRs, dAb_PD−L1/CD24_-mPDA NPs, and dAb_PD−L1/CD24_-mPDA@CuO_2_ NRs

For preparation of Ab_PD−L1_-mPDA@CuO_2_ NRs or Ab_CD24_-mPDA@CuO_2_ NRs, mPDA@CuO_2_ NRs were dispersed in 200 µL of pH 8.5 0.05 M Tris buffer. Subsequently, 20 µL of anti-PD-L1 or 20 µL of anti-CD24 antibodies were added to the solution containing mPDA@CuO_2_ NRs. The mixture was gently shaken overnight at 4 °C. To remove unbound antibodies, the samples were washed three times with DI-H_2_O through centrifugation at 12,000 rpm.

For preparation of dAb_PD−L1/CD24_-mPDA NPs or dAb_PD−L1/CD24_-mPDA@CuO_2_ NRs, mPDA NPs or mPDA@CuO_2_ NRs were dispersed in 200 µL of pH 8.5 0.05 M Tris buffer. Subsequently, 10 µL of anti-PD-L1 and 10 µL of anti-CD24 antibodies were added to the solution containing mPDA NPs or mPDA@CuO_2_ NRs. The mixture was gently shaken overnight at 4 °C. To remove unbound antibodies, the samples were washed three times with DI-H_2_O through centrifugation at 12,000 rpm. This process yielded dAb_PD−L1/CD24_-mPDA NPs or dAb_PD−L1/CD24_-mPDA@CuO_2_ NRs. The conjugation efficiency was determined by measuring the antibody concentration in the supernatant using ELISA.

### Apparatus

The morphology of CuO_2_, mPDA NPs, and mPDA@CuO_2_ NRs was characterized by transmission electron microscopy (TEM, H-7800, Hitachi), and the elemental composition of materials were characterized by energy dispersive spectroscopy on the scanning electron microscope (EDS-SEM, SU8220, Hitachi). The zeta potential and dispersion stability of materials were determined by dynamic light scattering (DLS, SZ-100, HORIBA, Japan). The catalyzed activity of mPDA@CuO_2_ NRs was analyzed by UV/VIS/NIR spectroscopy (MODEL V-700, JASCO, Japan).

### Studies of catalytic performance

The pH-triggered release of CuO_2_ from mPDA@CuO_2_ NRs for H_2_O_2_ catalysis was first investigated. For this purpose, 100 µL of H_2_O_2_ (1 mM) was mixed with 850 µL of mPDA@CuO_2_ NRs solution at pH values adjusted to 5.5 and 7.4. The mixture was incubated at 25 °C for 15–60 min. Afterward, 50 µL of TMB was added to the solution, and the absorbance intensity at 650 nm was measured using UV/VIS/NIR spectroscopy to determine the pH-dependent release efficiency of mPDA@CuO_2_ NRs.

Furthermore, to evaluate the catalytic performance of mPDA@CuO_2_ NRs, the mPDA@CuO_2_ NRs were pretreated in an acidic solution (pH 5.5) for 1–48 h. Then 850 µL of the pretreated mPDA@CuO_2_ NRs was mixed with 100 µL of H_2_O_2_ at different concentrations (0.1, 1.0, and 10 mM) at 25 °C for 1 min. Subsequently, 50 µL of TMB was added to the solution, and the absorbance intensity at 650 nm was measured using UV/VIS/NIR spectroscopy (MODEL V-700, JASCO, Japan) to determine the catalytic activity of mPDA@CuO_2_ NRs.

### In vitro cell studies

In this study, two TNBC cell lines, 4T1 (mouse) and MDA-MB-468 (human), were utilized. The cells were cultured in Dulbecco’s modified Eagle’s medium (DMEM) supplemented with 2.2 mg/mL sodium carbonate, 10% fetal bovine serum (FBS), and 50 µg/mL each of gentamicin, penicillin, and streptomycin. Before seeding into experimental wells, the cells were harvested using a 0.05% trypsin-ethylenediaminetetraacetic acid (EDTA) solution and washed three times with PBS buffer (pH = 7.4). TNBC cells were seeded at a density of 1.5 × 10^4^ cells per well in 96-well plates and cultured for 24 h. Subsequently, different concentrations (12.5, 25, 50, 100, and 200 µg/mL) of materials, namely mPDA NPs, dAb_PD−L1/CD24_-mPDA NPs, mPDA@CuO_2_ NRs, and dAb_PD−L1/CD24_-mPDA@CuO_2_ NRs, were added to the cells, followed by incubation for an additional 24 h. After 24 h, the culture medium was removed, and the cells were incubated with 120 µL of XTT solution for 2 h. Following this, 100 µL of XTT solution from each well was transferred to a separate 96-well counting plate. The cell viability of 4T1 or MDA-MB-468 cells was determined by measuring the optical density (OD) at 490 nm using a SpectraMax M2 microtiter plate reader.

To simulate a realistic tumor environment and investigate the generation of excessive •OH as the H_2_O_2_ concentration increased, 4T1 cells were cultured at a density of 1 × 10^4^ cells per well in U-end 96-well plates for 72 h to form spheroid 3D cultures. This step aimed to confirm the CuO_2_ in mPDA@CuO_2_ NRs as a source of excessive •OH production. The formed 4T1 tumor spheres were subsequently transferred to Transwell inserts. The wells were then supplemented with medium containing mPDA NPs, mPDA@CuO_2_ NRs, Ab_CD24_-mPDA@CuO_2_ NRs, and Ab_PD−L1_-mPDA@CuO_2_ NRs at a concentration of 100 µg/mL, respectively. Following a 48-h incubation period, the appearance and morphology of the 4T1 tumor cell spheroids were examined using inverted fluorescence microscopy (Nikon eclipse Ti2, Japan). This evaluation aimed to assess the efficiency of chemodynamic therapy (CDT) for 4T1 tumor cell spheroids.

To investigate the activation of CD8 + T cells, freshly isolated CD8 + T cells from WT C57BL/6 mice were co-cultured with 4T1 cancer cells in 12-well plates for 48 h. Prior to co-culturing, the 4T1 cells were pretreated with dAb_PD−L1/CD24_-mPDA NPs for 6 h at 37 °C. A blank control consisting of the co-culture system with only medium was included. After 48 h of treatment, the culture supernatants from each group were individually collected to analyze the levels of interferon-gamma (IFN-γ).

### ROS generation assay

The DCFDA/H2DCFDA - Cellular ROS Assay Kit was employed to assess the generation of free radicals upon the addition of mPDA@CuO_2_ NRs to cells. 4T1 cells were cultured in 12-well plates at a density of 5 × 10^4^ cells per well and incubated for 24 h. Subsequently, the original culture medium was removed, and mPDA@CuO_2_ NRs were resuspended in DMEM culture medium containing 1 × 10^− 4^ M of H_2_O_2_ for an additional 24-h incubation period. Following this, the cells were washed once with PBS, and 500 µL of DCFDA solution (2.5 µM) was added for 45 min. After two additional washes with PBS, the cells were examined using inverted fluorescence microscopy (Nikon eclipse Ti2, Japan) for monitoring purposes.

### Target efficiency of dual antibody-aided mesoporous nanoreactors

4T1 cells were seeded at a density of 5 × 10^5^ cells in 12-well plates. Following 24 h of incubation, the original culture medium was replaced with 1 mL of fresh DMEM medium containing mPDA NPs, Ab_CD24_-mPDA NPs, or Ab_PD−L1_-mPDA NPs at a final concentration of 100 µg/mL. After a 2-h incubation period, the residual materials were removed, and the cells were subjected to 10 washes with PBS. The cells were subsequently observed and recorded using inverted fluorescence microscopy.

### Anti-tumour effect in vivo

All animal experiments conducted in this study were approved by the Institutional Animal Care and Use Committee of China Medical University, Taiwan, and adhered to the guidelines for experimental animal care (IACUC NO. CMUIACUC-2021-109-1). The mice were housed at a room temperature of 26 °C. Female C57BL/6J mice weighing approximately 25–30 g (5–6 weeks old) were obtained from BioLASCO (Taipei, Taiwan) and were used to validate the effectiveness of the proposed approach. Prior to the commencement of the experiment, the mice were acclimatized for a minimum of two weeks. To establish the tumor models, 4T1 cells (1 × 10^5^ cells in 50 µL DMEM) were subcutaneously implanted into the mice. Tumor size was measured using a caliper every 2–3 days, and mice with tumor sizes reaching 100 mm^3^ were selected for the experimental studies.

The 4T1 tumor-bearing mice were randomly assigned to five groups, with 8 mice per group: (1) saline (control group), (2) mPDA NPs, (3) dAb_PD−L1/CD24_-mPDA NPs, (4) mPDA@CuO_2_ NRs, and (5) dAb_PD−L1/CD24_-mPDA@CuO_2_ NRs. A 50-µL suspension of the respective materials (2 mg/mL) was intratumorally injected into the tumor-bearing mice. The mice received treatment on day 7, day 9, day 11, day 14, and day 17 after the implantation of 4T1 tumor cells.

Tumor size and mouse body weight were recorded every other day, and tumor volume was calculated using the following formula:

$${\text{Volume}} = Tumor\,length \times \frac{{Tumor\,widt{h^2}}}{2}$$

### Histology

Mice were sacrificed after 4 days of treatment with mPDA@CuO_2_ NRs or dAb_PD−L1/CD24_-mPDA@CuO_2_ NRs, and tumor tissues were collected and stained with IFN-γ or CD68 to evaluate the ability of Ab_PD−L1/CD24_-mPDA@CuO_2_ NRs for T cells and CD68 + infiltrating macrophages reactivation observed under a digital microscope (TissueFAXS PLUS + HistoQuest).

### Statistical analysis

The data are expressed as the mean ± standard deviation (S.D.) on the basis of at least three independent experiments. Statistical analysis was performed using Student’s t-test. Differences were considered to be statistically significant for a *p value < 0.05.

## Results and discussions

### Characterization of mPDA@CuO_2_ NRs

**Fig. 1:**
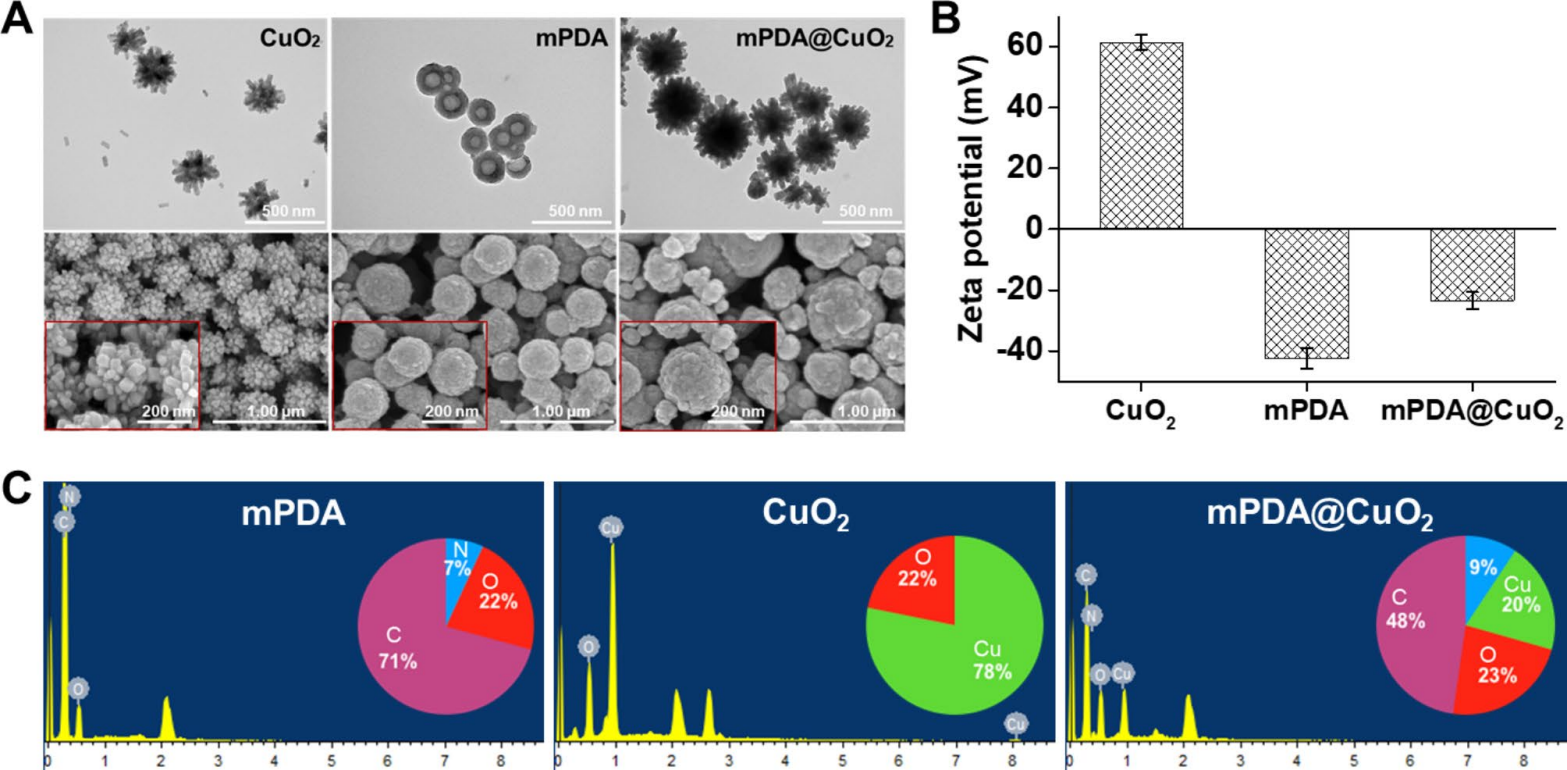
Characterization of synthesized materials. **(A)** Transmission electron microscopy (TEM) images and scanning electron microscopy (SEM) images of CuO_2_ clusters, mPDA NPs, and mPDA@CuO_2_ NRs. **(B)** Zeta potential of CuO_2_ clusters, mPDA NPs, and mPDA@CuO_2_ NRs. The values are expressed as means ± SD (n = 3). **(C)** Elemental analysis of CuO_2_ clusters, mPDA NPs, and mPDA@CuO_2_ NRs

CuO_2_ clusters were initially synthesized through the reaction of copper(II) chloride (CuCl_2_), H_2_O_2_, and alkaline Tris buffer (pH 8.5) at room temperature for 30 min. The resulting CuO_2_ clusters served as a template for the preparation of mPDA@CuO_2_ NRs. Transmission electron microscopy (TEM) and scanning electron microscopy (SEM) images revealed that the CuO_2_ clusters exhibited a distinctive urchin-like shape, with an average particle size of 260.4 ± 15 nm, indicating their composition of multiple CuO_2_ clusters (Fig. 1A, left). On the other hand, mPDA displayed a spherical, hollow, and smooth morphology, with an average particle size of 184.0 ± 10 nm (Fig. 1A, middle). Upon the formation of mPDA@CuO_2_ NRs, the central portion of the original CuO_2_ clusters was coated with mPDA, while the uncoated outer part retained a meteor hammer shape, with an average particle size of 283 ± 16 nm (Fig. 1A, right). Zeta potential analysis demonstrated that the surface potential of CuO_2_ clusters was 61.3 ± 2.5 mV, which changed to -23.5 ± 2.8 mV upon coating with mPDA (-42.5 ± 3.6 mV). This change can be attributed to the deprotonation of phenol groups in mPDA, resulting in a negatively charged surface at neutral pH (Fig. 1B). These results confirm the successful coating of mPDA onto the surface of CuO_2_ clusters, leading to the formation of mPDA@CuO_2_ NRs. Energy-dispersive X-ray spectroscopy (EDS) further supported the successful preparation of mPDA@CuO_2_ NRs. The elemental composition analysis indicated that mPDA predominantly consisted of carbon (C) (71%), oxygen (O) (22%), and nitrogen (N) (7%), while CuO_2_ clusters was primarily composed of copper (Cu) (78%) and oxygen (O) (22%). After coating mPDA onto CuO_2_ clusters, the observed elements were carbon (C) (48%), nitrogen (N) (9%), copper (Cu) (20%), and oxygen (O) (23%), confirming the successful preparation of mPDA@CuO_2_ NRs with approximately 20% of CuO_2_ clusters remaining uncoated with mPDA (Fig. 1C).

### Acid-induced •OH generation from mPDA@CuO_2_ NRs

**Fig. 2:**
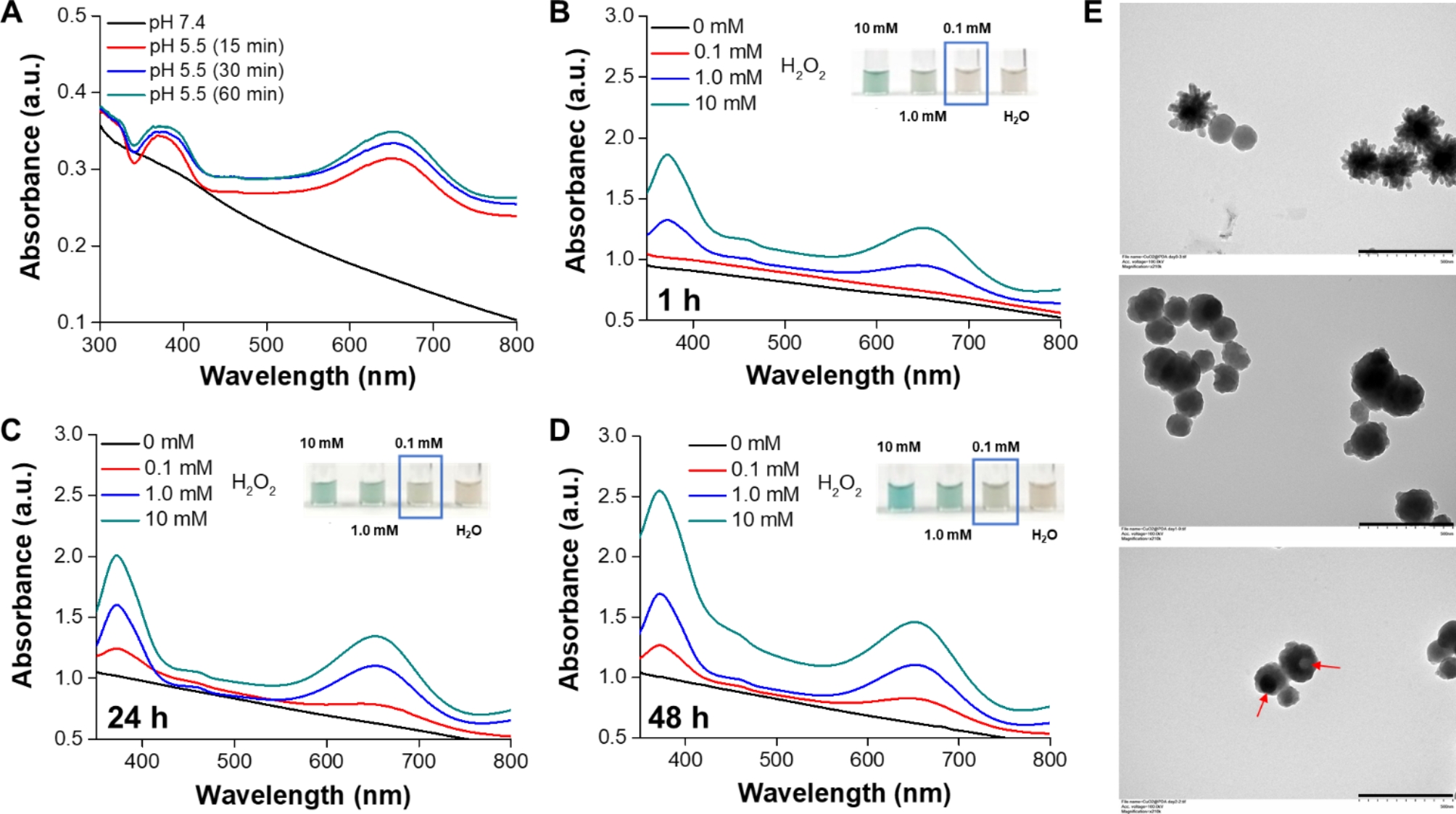
**(A)** Colorimetric detection of •OH generated by mPDA@CuO_2_ NRs at different pH values based on the TMB assay. UV-vis spectra and photographs (inset) of TMB aqueous solution incubated with different concentrations of H_2_O_2_ in the presence of mPDA@CuO_2_ NRs (100 µg/mL) pretreated in an acidic solution for **(B)** 1 h, **(C)** 24 h, and **(D)** 48 h. **(E)** TEM images of mPDA@CuO_2_ NRs (*top*), mPDA@CuO_2_ NRs treated in an acidic solution for 1 h (*middle*), and mPDA@CuO_2_ NRs treated in an acidic solution for 24 h (*bottom*). Scale bar = 500 nm

In an acidic environment, the dissociation of CuO_2_ clusters into Cu^2+^ and H_2_O_2_, along with the subsequent Fenton-type reaction between these dissociation products, generates •OH for cancer chemodynamic therapy (CDT). However, the rapid decomposition of CuO_2_ clusters to Cu^2+^ and H_2_O_2_ in an acidic environment limits the duration of CDT. From the results in Fig. S1, a discernible color change in TMB to a blue-green hue, exhibiting a distinct absorbance peak at approximately 650 nm, was observed when the CuO_2_ NRs were incubated in an acidic solution for 30 min, compared to mPDA@CuO_2_ NRs. This suggests that the coating of mPDA can impede the rapid decomposition of CuO_2_ NRs. To address this, we developed mPDA-coated CuO_2_ NPs (mPDA@CuO_2_ NRs) with acid-triggered degradation capability to prevent their rapid decomposition in the acidic tumor microenvironment. To demonstrate the acid-triggered H_2_O_2_ self-supplying ability of mPDA@CuO_2_ NRs, we suspended the NRs in an acidic solution (pH 5.5) without the addition of H_2_O_2_ and incubated them for different time intervals. As expected, a noticeable color change from TMB to a blue-green color with a distinct absorbance peak at approximately 650 nm was observed after 15 min of incubation under mild acidic (pH 5.5) conditions, but not under neutral (pH 7.4) conditions (Fig. 2A). These findings confirm that the mPDA@CuO_2_ NRs possess acid-responsive characteristics, allowing them to produce Fenton catalytic Cu^2+^ and H_2_O_2_ for self-supplying CDT in the acidic tumor microenvironment [29]. Subsequently, we performed an initial pretreatment of the mPDA@CuO_2_ NRs by resuspending them in an acidic solution (pH 5.5) for 1–48 h to etch away the outer CaO_2_ clusters layer and mPDA coating layer of the mPDA@CuO_2_ NRs. As a result, the spikes on the surface of mPDA@CuO_2_ NRs disappeared after 1 h incubation, causing a morphological transition from an urchin-like shape to a sphere-like shape (Fig. 2E, middle). The resulting mPDA@CuO_2_ NRs were then mixed with different concentrations (0–10 mM) of H_2_O_2_. The results depicted in Fig. 2B demonstrated that the mPDA@CuO_2_ NRs were capable of generating sufficient •OH to oxidize TMB only in the presence of high concentrations of H_2_O_2_ (above 1 mM). No significant peak at 650 nm was observed when the H_2_O_2_ concentration was below 1 mM during the initial 1 h. Notably, after 24 h of incubation in the acidic solution (pH 5.5), the mPDA structure gradually loosened, leading to the formation of a hollow sphere-like shape (Fig. 2E, bottom). This structural change allowed for more CuO_2_ clusters to decompose into Cu^2+^ and H_2_O_2_ in the acidic environment, resulting in sufficient •OH generation to oxidize TMB even at lower H_2_O_2_ concentrations (0.1 mM, mimicking the tumor microenvironment) (Fig. 2C&D). The inner CuO_2_ clusters layer underwent Fenton reaction, generating •OH. The absorption peaks at 370/650 nm increased with the H_2_O_2_ concentration and the duration of mPDA immersion in the solution, indicating that •OH generation was directly proportional to the H_2_O_2_ concentration. This observation is likely due to the slow loosening of the mPDA structure in a neutral environment and its rapid loosening in an acidic environment. These results demonstrate that the mPDA coating effectively prevents the rapid decomposition of CuO_2_ clusters into Cu^2+^ and H_2_O_2_ in a neutral environment, thus reducing potential side effects in normal tissues.

### In vitro ROS generation and CDT efficacy

**Fig. 3:**
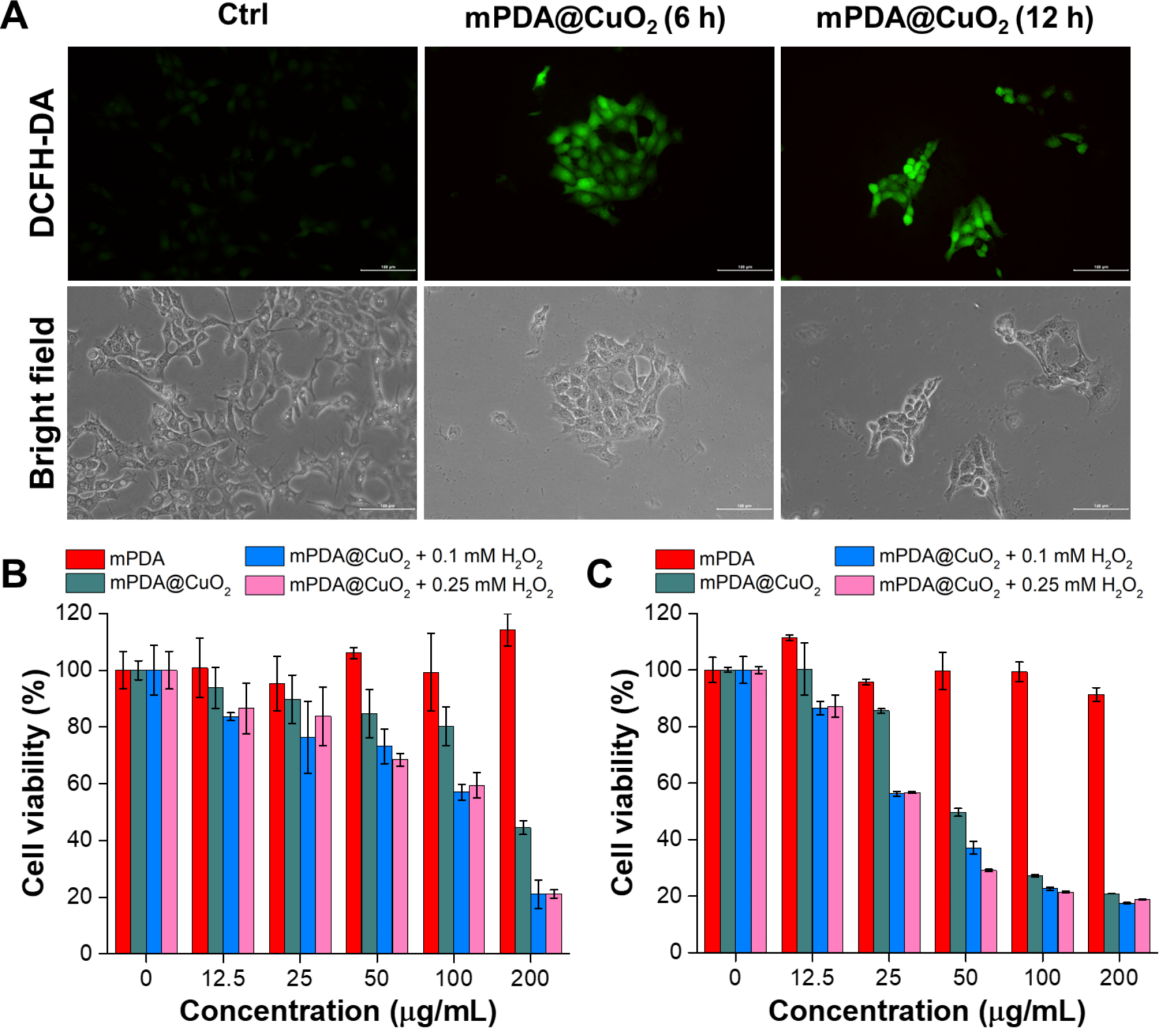
**(A)** Fluorescence and bright-field images of DCFH-DA-stained 4T1 cancer cells after exposure to mPDA@CuO_2_ NRs for 6 and 12 h. The scale bar represents 100 μm. **(B)** In vitro CDT potency of mPDA@CuO_2_ NRs after 24 h of incubation with MDA-MB-468 cells in the presence of different concentrations of H_2_O_2_. The values are expressed as means ± SD (n = 8). **(C)** In vitro CDT potency of mPDA@CuO_2_ NRs after 24 h of incubation with 4T1 cells in the presence of different concentrations of H_2_O_2_. The values are expressed as means ± SD (n = 8)

pH-sensitive mPDA@CuO_2_ NRs can undergo decomposition within cancer cells following endocytosis, leading to a Fenton-like reaction between the released Cu^2+^ and H_2_O_2_ in the acidic environment of endosomes, resulting in the generation of •OH. To evaluate the production of •OH by mPDA@CuO_2_ NRs at the cellular level, we utilized 2’,7’-dichlorofluorescin diacetate (DCFH-DA) as a fluorescent indicator of reactive oxygen species (ROS). Increased fluorescence intensity indicates a higher level of •OH generation. The results depicted in Fig. 3A demonstrate that 4T1 cancer cells incubated with mPDA@CuO_2_ NRs for 6 h exhibited significantly higher green fluorescence (Fig. 3A, middle) compared to the untreated control group (Fig. 3A, left). Moreover, the ROS-associated green fluorescence signal was still observable even after incubating the cells with mPDA@CuO_2_ NRs for over 12 h (Fig. 3A, right). This observation suggests that mPDA@CuO_2_ NRs efficiently generate •OH within cancer cells, allowing for a sustained two-stage Fenton-like reaction and longer-lasting chemodynamic therapy (CDT).

Subsequently, we quantitatively assessed the in vitro CDT efficiency of mPDA@CuO_2_ NRs against cancer cells (MDA-MB-468 cells and 4T1 cells) using the XTT assay. As depicted in Fig. 3B, MDA-MB-468 cells treated with 25 µg/mL of mPDA@CuO_2_ NRs for 24 h exhibited a cell viability of approximately 85.6%. However, when an additional H_2_O_2_ concentration of 0.1 mM was introduced into the culture medium, the cell viability significantly decreased to 56.2%. This decrease in cell viability can be attributed to the fact that the low concentration of mPDA@CuO_2_ NRs (25 µg/mL) was insufficient to generate an adequate amount of H_2_O_2_, resulting in an inadequate production of •OH. Interestingly, when the concentration of mPDA@CuO_2_ NRs was increased to 50 µg/mL, more MDA-MB-468 cells were killed. Intriguingly, mPDA@CuO_2_ NRs exhibited lower toxicity towards 4T1 cells, which can be attributed to their rapid proliferation rate (Fig. 3C). Nonetheless, even at a concentration of 200 µg/mL, the cell viability of 4T1 cells decreased to 44.5%. Furthermore, when an additional H_2_O_2_ concentration of 0.1 mM was introduced, the cell viability further decreased to 20.9%. These results indicate that MDA-MB-468 cells were more sensitive to mPDA@CuO_2_ NRs, with an IC_50_ value of 51.9 µg/mL, while 4T1 cells had an IC_50_ value of 190.7 µg/mL. Moreover, when H_2_O_2_ was introduced at a concentration similar to the tumor microenvironment (0.1 mM), the IC_50_ values significantly decreased to 25.6 µg/mL for MDA-MB-468 cells and 106.9 µg/mL for 4T1 cells. These findings further confirm that mPDA@CuO_2_ NRs, which serve as enhanced chemodynamic nanoagents with self-supplied H_2_O_2_, exhibit potent anticancer activity. Moreover, their cytotoxicity efficiency against TNBC cells is concentration-dependent.

### Investigation of PD-L1 and CD24 expression in TNBC cells

**Fig. 4:**
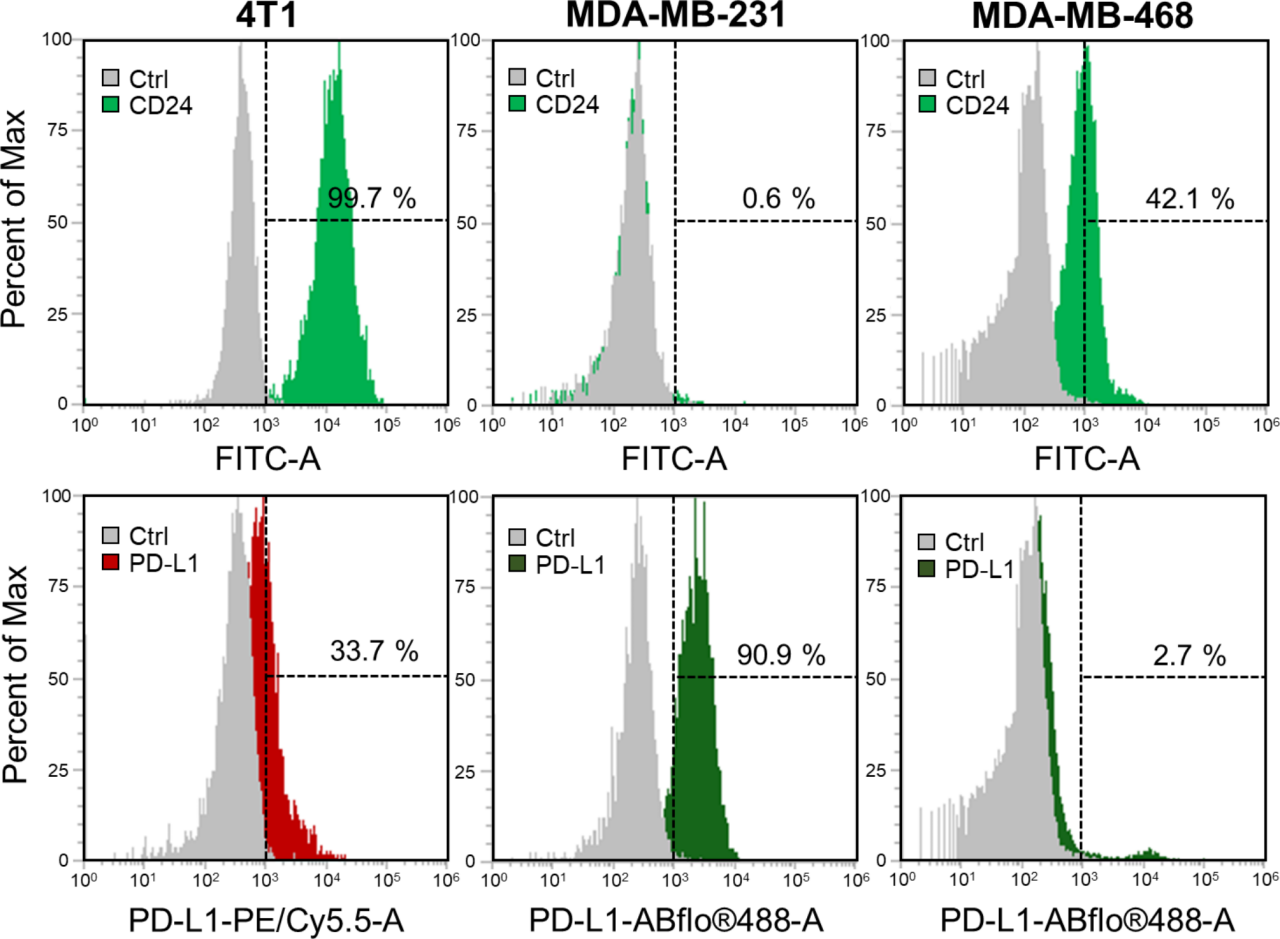
Flow cytometry analysis of PD-L1 and CD24 protein expression on the cell membrane of 4T1, MDA-MB-231, and MDA-MB-468 cells

In this study, our objective is to combine chemodynamic therapy (CDT) with checkpoint blockade immunotherapy (CBIT) for the treatment of triple-negative breast cancer (TNBC). To assess the expression levels of immune checkpoint proteins in TNBC cells, specifically PD-L1 and CD24, we conducted flow cytometry analysis on two types of human TNBC cells (MDA-MB-231, and MDA-MB-468) as well as one mouse TNBC cell line (4T1 cells). The data presented in Fig. 4 demonstrate that, among the examined cell lines, only 4T1 cells exhibit concurrent expression of both PD-L1 and CD24, with the potential for CD24 expression reaching up to 99.7%. Conversely, MDA-MB-231 and MDA-MB-468 cells exclusively express either PD-L1 or CD24. More specifically, MDA-MB-231 cells display a high expression level of PD-L1 (90.9%) without notable expression of CD24 (0.6%). Although MDA-MB-468 cells do manifest expression of both CD24 (42.1%) and PD-L1 (2.7%), the level of PD-L1 expression is extremely low, verging on negligible. From the results, it is evident that not all cells will express both PD-L1 and CD24 simultaneously. Based on these findings, we endeavored to immobilize Ab_PD−L1_ and Ab_CD24_ concurrently on the surface of mPDA@CuO_2_ NRs. This led to the creation of dAb_PD−L1/CD24_-mPDA@CuO_2_ NRs, allowing for a broader and more comprehensive application of CBIT for TNBC. This strategy facilitates the integration of CDT and dual CBIT in treating TNBC.

### Quantification of Ab_PD−L1_ and Ab_CD24_ immobilization

**Fig. 5:**
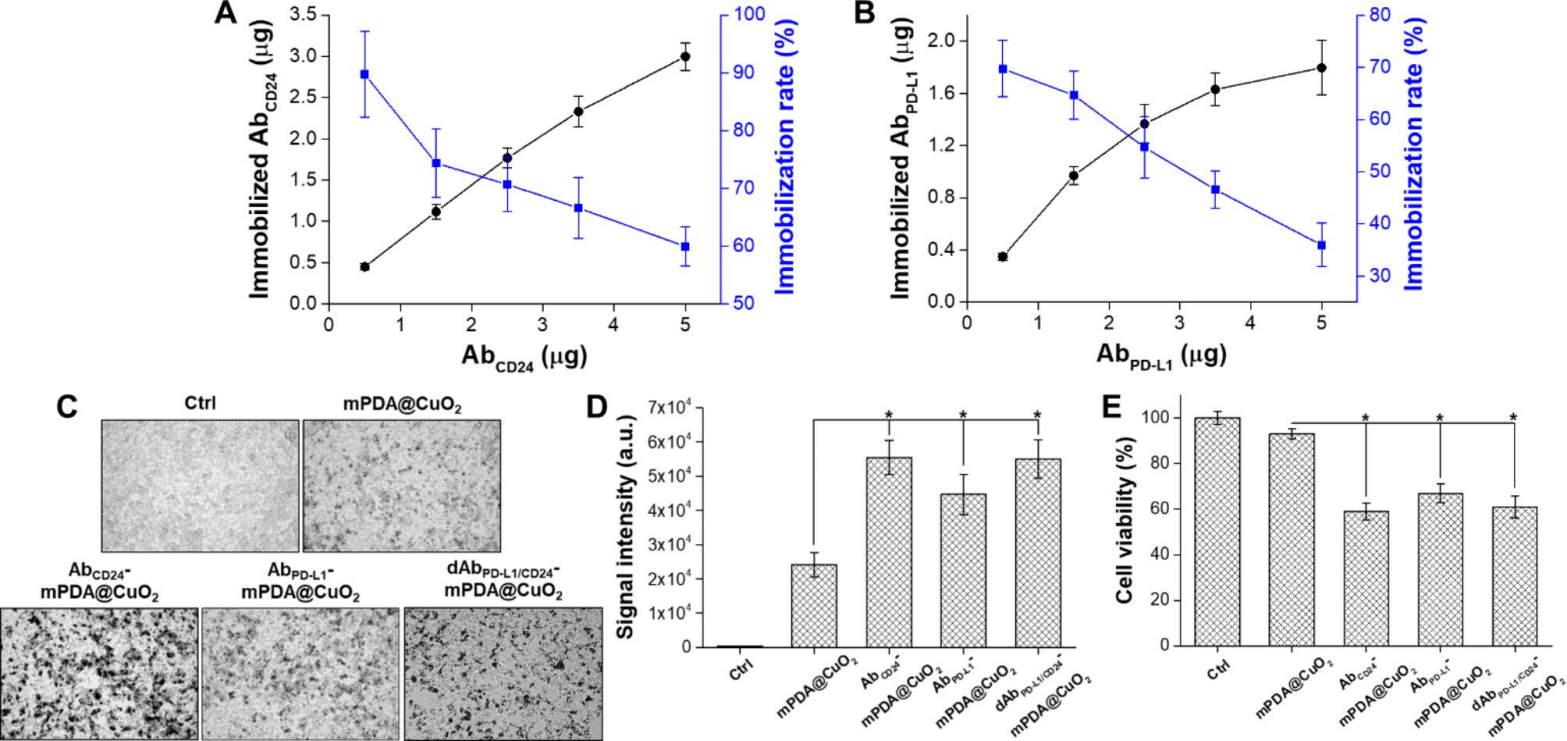
**(A)** Analysis of the optimal immobilization rate of Ab_CD24_ on mPDA@CuO_2_ NRs using ELISA. **(B)** Analysis of the optimal immobilization rate of Ab_PD−L1_ on Ab_CD24_-mPDA@CuO_2_ NRs using ELISA. **(C)** Bright-field images of 4T1 cells treated with PBS (Ctrl), mPDA@CuO_2_ NRs, Ab_CD24_-mPDA@CuO_2_ NRs, Ab_PD−L1_-mPDA@CuO_2_ NRs, and dAb_PD−L1/CD24_-mPDA@CuO_2_ NRs. The black dots indicate mPDA@CuO_2_ NRs. **(D)** The data are presented as the mean intensity of black dots, which was calculated from the images in **(C)**. The values are expressed as means ± SD (n = 3). Asterisks indicate a significant difference between the mPDA@CuO_2_ NRs and Ab_CD24_-mPDA@CuO_2_ NRs, Ab_PD−L1_-mPDA@CuO_2_ NRs, dAb_PD−L1/CD24_-mPDA@CuO_2_ NRs groups (Student’s t-test, *p ≤ 0.05). **(E)** The effect of residual mPDA@CuO_2_ NRs, Ab_CD24_-mPDA@CuO_2_ NRs, Ab_PD−L1_-mPDA@CuO_2_ NRs, and dAb_PD−L1/CD24_-mPDA@CuO_2_ NRs on 4T1 cell viability measured by XTT assay after an additional 24 h of incubation. The values are expressed as means ± SD (n = 3). Asterisks indicate a significant difference between the mPDA@CuO_2_ NRs and Ab_CD24_-mPDA@CuO_2_ NRs, Ab_PD−L1_-mPDA@CuO_2_ NRs, dAb_PD−L1/CD24_-mPDA@CuO_2_ NRs groups (Student’s t-test, *p ≤ 0.05)

The quantities of Ab_PD−L1_ and Ab_CD24_ bound to mPDA@CuO_2_ NRs were determined by analyzing the unbound antibodies in the supernatant using an ELISA method. The amount of Ab_CD24_ immobilized on 100 µg of mPDA@CuO_2_ NRs increased with higher concentrations of added Ab_CD24_. At 5 µg of added Ab_CD24_, approximately 3.0 ± 0.2 µg of Ab_CD24_ was bound to 100 µg of mPDA@CuO_2_ NRs, resulting in a conjugation rate of 59.9 ± 3.4% (Fig. 5A). On the other hand, the quantity of immobilized Ab_PD−L1_ reached saturation (1.6 ± 0.1 µg Ab_PD−L1_/100 µg mPDA@CuO_2_ NRs) at 3.5 µg of added Ab_PD−L1_, with a conjugation rate of 46.6 ± 3.6%. Although the amount of immobilized Ab_PD−L1_ could be increased to 1.8 ± 0.2 µg with 5 µg of added Ab_PD−L1_, the conjugation rate significantly decreased to 35.9 ± 4.2% (Fig. 5B). The reduced conjugation rate might be due to the majority of the surface area being occupied by Ab_CD24_. This could possibly be attributed to the higher affinity of Ab_CD24_ with mPDA, resulting in a lower conjugation rate for Ab_PD−L1_ compared to Ab_CD24_. Taken together, these results demonstrate that approximately 3.0 ± 0.2 µg of Ab_CD24_ and 1.6 ± 0.1 µg of Ab_PD−L1_ can be immobilized on 100 µg of mPDA@CuO_2_ NRs.

### In vitro cell targeting efficacy and cytotoxicity

We examined the binding efficiency of mPDA NPs, Ab_CD24_-mPDA@CuO_2_ NRs, and Ab_PD−L1_-mPDA@CuO_2_ NRs to 4T1 cells by incubating the materials with the cells for 2 h, followed by washing with fresh DMEM medium to remove unbound materials. As shown in Fig. 5C&D, Ab_CD24_-mPDA@CuO_2_ NRs (signal intensity: 5.6 × 10^4^), Ab_PD−L1_-mPDA@CuO_2_ NRs (signal intensity: 4.5 × 10^4^), and dAb_PD−L1/CD24_-mPDA@CuO_2_ NRs (signal intensity: 5.5 × 10^4^) all demonstrated significantly higher attachment to the cell membrane compared to mPDA@CuO_2_ NRs (signal intensity: 2.4 × 10^4^), with approximately 1.9 ~ 2.4-fold higher signal intensity. However, the amount of dAb_PD−L1/CD24_-mPDA@CuO_2_ NRs attached to the 4T1 cells was not significant higher than that of Ab_PD−L1_-mPDA@CuO_2_ NRs or Ab_CD24_-mPDA@CuO_2_ NRs. This is because their total antibody content is similar. Yet, since dAb_PD−L1/CD24_-mPDA@CuO_2_ NRs contain both Ab_PD−L1_ and Ab_CD24_, its advantage lies in its ability to target cells more effectively, as long as the cells express either PD-L1 or CD24. To investigate the impact of targeting on CDT, the 4T1 cells were further incubated in fresh DMEM containing 0.1 mM H_2_O_2_ at 37 °C for 24 h after removing unbound materials (Fig. 5E). The cell viability was 93 ± 2.3% for the mPDA@CuO_2_ NRs group, but significantly decreased to 59 ± 3.7% for the Ab_CD24_-mPDA@CuO_2_ NRs group, 63 ± 4.1% for the Ab_PD−L1_-mPDA@CuO_2_ NRs group, and 61 ± 4.7% for the dAb_PD−L1/CD24_-mPDA@CuO_2_ NRs group compared to the control group (treated with 0.1 mM H_2_O_2_). These results indicate that the modification of mPDA@CuO_2_ NRs with Ab_CD24_ and Ab_PD−L1_ effectively targets 4T1 cells, leading to enhanced CDT efficacy and blocking of CD24-Siglec-10 and PD-L1-PD1 signaling pathways.

**Fig. 6:**
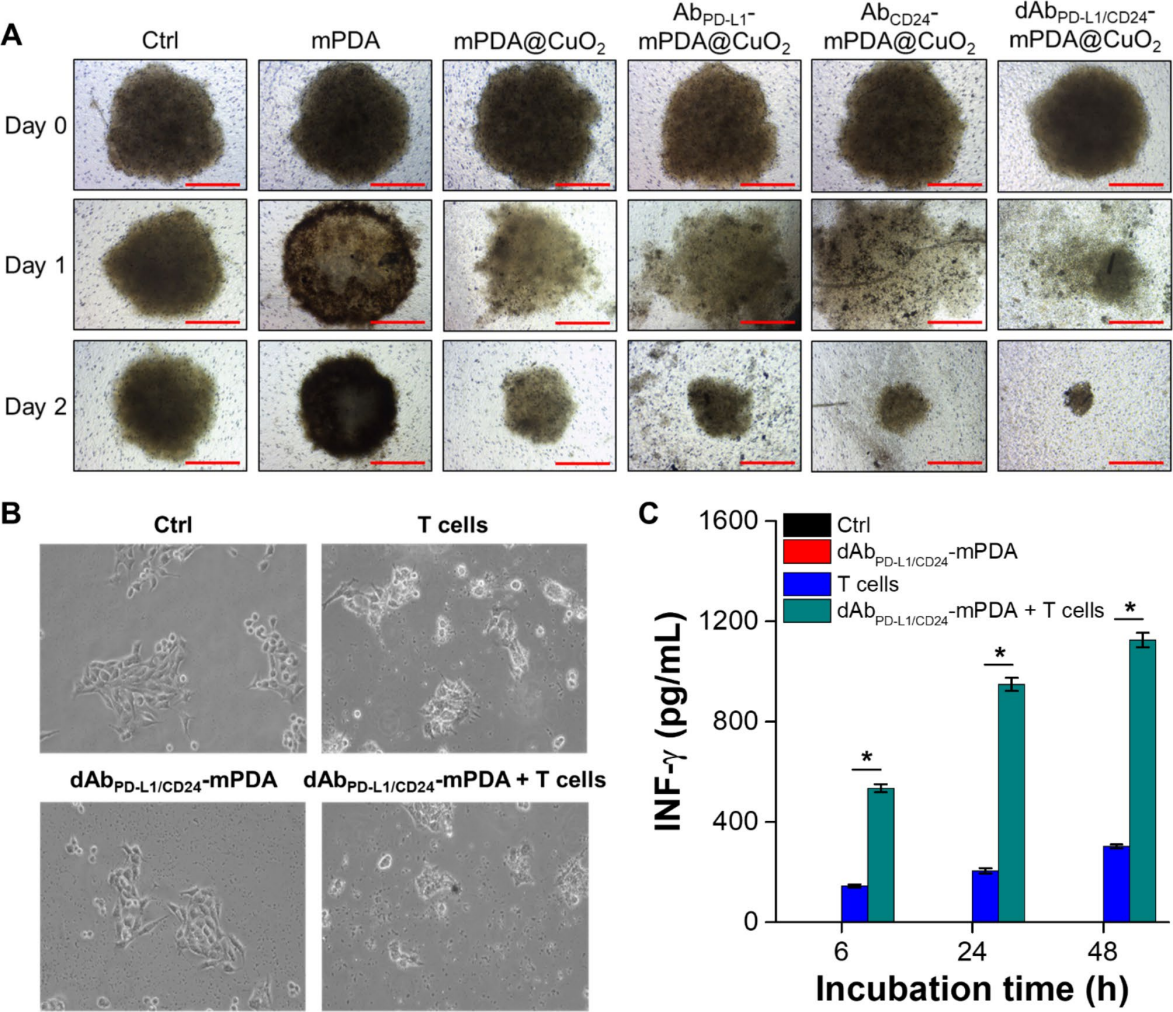
4T1 cells were cultured in an ultra-low attachment plate to form tumor spheroids, followed by treatment with different materials in the culture medium over time. **(A)** The volume of 4T1 tumor spheroids after treatment with PBS (Ctrl), mPDA NPs, mPDA@CuO_2_ NRs, Ab_PD−L1_-mPDA@CuO_2_ NRs, Ab_CD24_-mPDA@CuO_2_ NRs, and dAb_PD−L1/CD24_-mPDA@CuO_2_ NRs. Scale bar = 500 μm. **(B)** In vitro CBIT efficiency tested by blocking PD-L1–PD1 signaling between cancer cells and T cells using dAb_PD−L1/CD24_-mPDA NPs. **(C)** Generation of IFN-γ from T cells after co-culturing with 4T1 cells treated with dAb_PD−L1/CD24_-mPDA NPs. The values are expressed as means ± SD (n = 3). Asterisks indicate a significant difference between the T cells treated and dAb_PD−L1/CD24_-mPDA NPs + T cells treated groups (Student’s t-test, *p ≤ 0.05). Note: The absence of black and red bars is due to their values being extremely low, almost equivalent to 0

We further investigated the cell targeting and anticancer efficiency of mPDA NPs, mPDA@CuO_2_ NRs, Ab_CD24_-mPDA@CuO_2_ NRs, Ab_PD−L1_-mPDA@CuO_2_ NRs, and dAb_PD−L1/CD24_-mPDA@CuO_2_ NRs using a cultured three-dimensional tumor spheroid by employing 4T1 cells as a cell model. The 4T1 tumor spheroid was formed in an ultra-low attachment round-bottomed plate and then transferred to an agarose-coated flat-bottomed 96-well plate. The spheroids were pretreated with different materials for 6 h, and then transferred to a new well and cultured with fresh DMEM for an additional one or two days. As shown in Fig. 6A, after one day of incubation, the 4T1 tumor spheroid in the mPDA@CuO_2_ NRs, Ab_CD24_-mPDA@CuO_2_ NRs, Ab_PD−L1_-mPDA@CuO_2_ NRs, and dAb_PD−L1/CD24_-mPDA@CuO_2_ NRs groups began to disintegrate and appeared looser compared to the group treated with mPDA NPs. Furthermore, the volume of the 4T1 tumor spheroid treated with Ab_CD24_-mPDA@CuO_2_ NRs or Ab_PD−L1_-mPDA@CuO_2_ NRs was smaller than the control group and the mPDA@CuO_2_ NRs-treated group after two days of incubation. This indicates that Ab_CD24_-mPDA@CuO_2_ NRs or Ab_PD−L1_-mPDA@CuO_2_ NRs efficiently attached to the 4T1 tumor spheroid, leading to H_2_O_2_ self-supplying CDT. Notably, the volume of the 4T1 tumor spheroid treated with dAb_PD−L1/CD24_-mPDA@CuO_2_ NRs was the smallest (241.3 ± 23.8 μm) among the treatment groups (1002.7 ± 68.1 μm for mPDA NPs and 434.7 ± 105.8 μm for mPDA@CuO_2_ NRs) after two days of incubation (Fig. S2). This is likely due to the enhanced attachment of dAb_PD−L1/CD24_-mPDA@CuO_2_ NRs to the 4T1 tumor spheroid, as dAb_PD−L1/CD24_-mPDA@CuO_2_ NRs can bind to CD24 and PD-L1 on the cell membrane simultaneously, enabling highly efficient H_2_O_2_ self-supplying CDT on the surface of the 4T1 tumor spheroid.

### Study of in vitro CBIT efficiency

The expression level of PD-1/PD-L1 in tumor tissue is known to be associated with clinical outcomes, tumor metastasis, and overall survival in various cancers, including melanoma, breast cancer, and pancreatic cancer. In this study, we aimed to develop dAb_PD−L1/CD24_-mPDA@CuO_2_ NRs to enhance the immune cells’ ability to detect and eliminate cancer cells. To verify the efficacy of dAb_PD−L1/CD24_-mPDA@CuO_2_ NRs in checkpoint blockade immunotherapy (CBIT), we executed successful blockage of PD-L1 and CD24 on 4T1 cells. This was done by pretreating the cells with dAb_PD−L1/CD24_-mPDA@CuO_2_ NRs for 6 h and subsequently staining for residual, unblocked PD-L1 and CD24. The results revealed that approximately 20.2 ± 4.8% of CD24 and 38.5 ± 6.2% of PD-L1 on the 4T1 cell surface were effectively blocked by dAb_PD−L1/CD24_-mPDA@CuO_2_ NRs (Fig. S3). Following this, the cells were co-cultured with mouse T cells for an additional 48 h. The results shown in Fig. 6B revealed that the morphology of 4T1 cells exhibited slight differences after co-culturing with T cells compared to the control group and the group treated with dAb_PD−L1/CD24_-mPDA NPs alone. This can be attributed to the PD-L1-PD1 signaling between 4T1 cells and T cells, which transmits a “don’t eat me” signal to T cells [30]. However, when 4T1 cells were pretreated with dAb_PD−L1/CD24_-mPDA NPs to block PD-L1-PD1 signaling and reactivate T cells, the T cells successfully attacked and destroyed the 4T1 cells. Furthermore, we investigated the concentrations of IFN-γ secreted by T cells, as activated CD8 + cytotoxic T lymphocytes (CTLs) exert their antitumor effects by releasing IFN-γ, tumor necrosis factor-alpha (TNF-α), and other cytotoxins [31]. As depicted in Fig. 6C, only a minimal amount of IFN-γ (302.3 ± 8.1 pg/mL at 48 h of co-culture) was secreted when T cells were directly co-cultured with 4T1 cells. However, when T cells were co-cultured for 48 h with 4T1 cells that had been pretreated with dAb_PD−L1/CD24_-mPDA NPs, the concentration of secreted IFN-γ significantly increased to 1125.3 ± 28.9 pg/mL. These results confirm that dAb_PD−L1/CD24_-mPDA NPs effectively block PD-L1-PD1 signaling between 4T1 cells and T cells, reactivate T cells, and promote the secretion of sufficient IFN-γ to induce cancer cell death [32].

### dAb_PD−L1/CD24_-mPDA@CuO_2_ NRs inhibit Tumor growth in the tumor-bearing mice

**Fig. 7:**
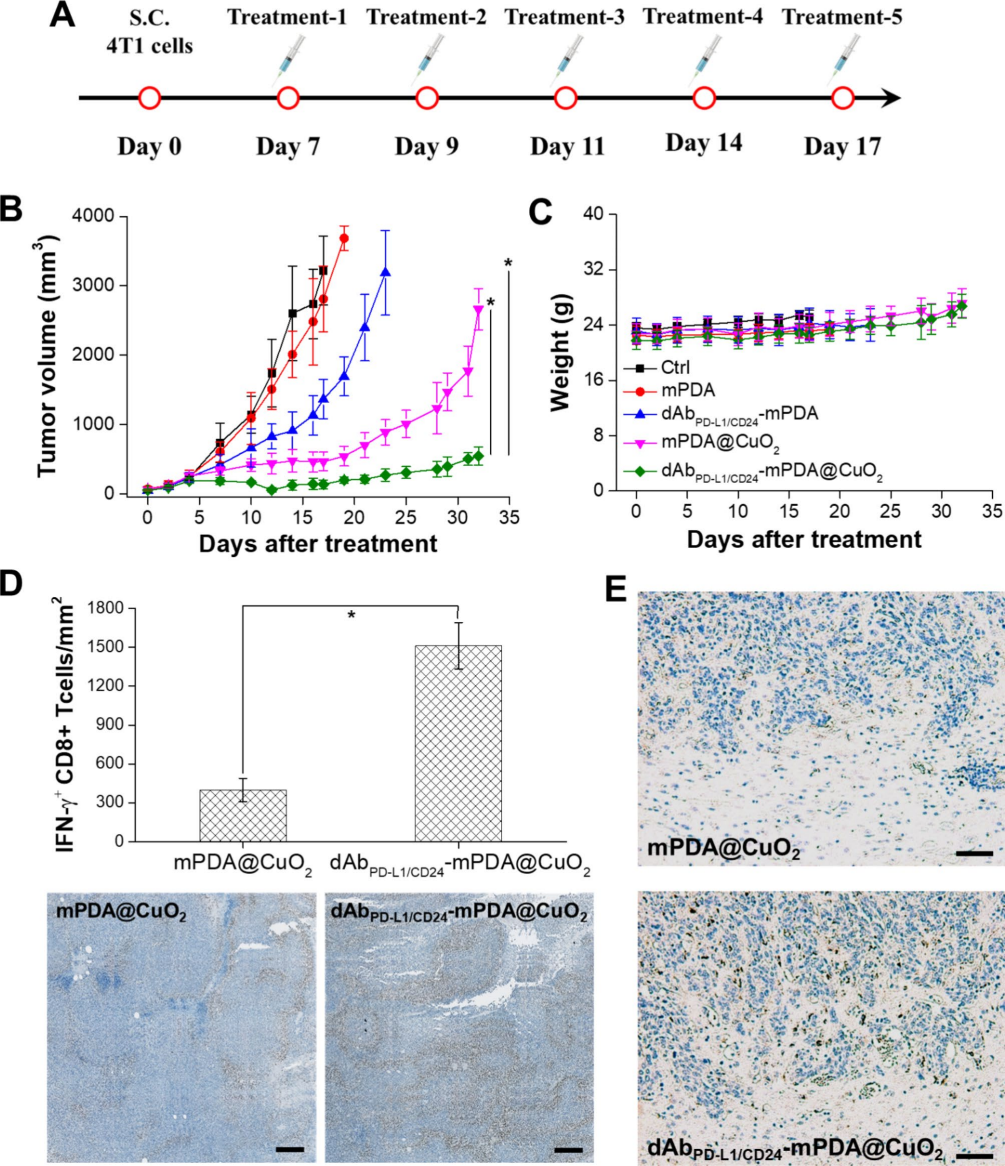
Anti-tumor effect of Ab_PD−L1/CD24_-mPDA@CuO_2_ NRs in vivo. **(A)** Treatment protocols assessing H_2_O_2_ self-supplying CDT + CBIT by intratumoral injection of dAb_PD−L1/CD24_-mPDA@CuO_2_ NRs. **(B)** Time-dependent tumor growth curves in 4T1 tumor-bearing mice after various treatments with intratumoral injections. The values are expressed as means ± SD (n = 8). Asterisks indicate a significant difference between the Ab_PD−L1/CD24_-mPDA@CuO_2_ NRs treated group and the Ab_PD−L1/CD24_-mPDA NPs and mPDA@CuO_2_ NRs treated groups (Student’s t-test, *p ≤ 0.05). **(C)** Body weight of 4T1 tumor-bearing mice in different groups after treatments. The values are expressed as means ± SD (n = 8). **(D)** Protein expression level of IFN-γ in tumor tissues after treatment to block the PD-L1–PD1 signaling between cancer cells and T cells using Ab_PD−L1/CD24_-mPDA@CuO_2_ NRs (the bottom inset shows a corresponding digital photo of immunohistochemistry). The values are expressed as means ± SD (n = 3). Asterisks indicate a significant difference (Student’s t-test, *p ≤ 0.05). Scale bar = 200 μm. **(E)** Immunohistochemical analysis of immune responses, focusing on CD68 (infiltrating macrophages) in tumor tissues from mice treated with mPDA@CuO_2_ NRs and dAb_PD−L1/CD24_-mPDA@CuO_2_ NRs. Scale bar = 500 μm

The therapeutic efficacy of dAb_PD−L1/CD24_-mPDA@CuO_2_ NRs in TNBC was evaluated by monitoring tumor size. When the tumor volume reached approximately 100 mm^3^, mice were treated intratumorally with PBS (control), blank mPDA NPs, dAb_PD−L1/CD24_-mPDA NPs, mPDA@CuO_2_ NRs, and dAb_PD−L1/CD24_-mPDA@CuO_2_ NRs (approximately 4 mg/kg) on day 7, day 9, day 11, day 14, and day 17 after the implantation of 4T1 tumor cells to assess the antitumor efficacy (Fig. 7A).

As depicted in Fig. 7B, treatment with mPDA NPs did not exhibit significant anti-tumor efficiency due to the lack of H_2_O_2_ self-supplying CDT and T-cell activation. However, at day 17, mice treated with dAb_PD−L1/CD24_-mPDA NPs showed a reduction in tumor volume (1362.9 ± 284.8 mm^3^) compared to the control group (3218.5 ± 498.6 mm^3^) and mPDA NPs treated group (2809.5 ± 477.5 mm^3^). This reduction can be attributed to the binding of dAb_PD−L1/CD24_-mPDA NPs to CD24 and PD-L1 molecules on tumor cells, which can target cytotoxic T lymphocytes against tumor cells and induce cytokine production (e.g., IFN-γ). However, after 17 days of CBIT treatment, the tumor began to grow rapidly, possibly due to the incomplete blockade of PD-L1 on the tumor cell membrane by dAb_PD−L1/CD24_-mPDA NPs. Therefore, combining CBIT with other treatment strategies (e.g., chemotherapy, photothermal therapy, and CDT) is necessary to achieve complete ablation of TNBC. Furthermore, Fig. 7B demonstrates that tumor growth could be effectively inhibited (463.1 ± 134.9 mm^3^ at day 17) when mice received mPDA@CuO_2_ NRs for H_2_O_2_ self-supplying CDT. However, tumor recurrence was observed after 21 days of H_2_O_2_ self-supplying CDT treatment, likely due to the revival of non-affected tumor cells caused by insufficient generation of •OH.

In this study, we integrated CBIT with H_2_O_2_ self-supplying CDT to achieve better tumor inhibition. Our findings revealed that dAb_PD−L1/CD24_-mPDA@CuO_2_ NRs inhibited tumor growth in most mice, and no significant tumor recurrence was observed until day 32 (545.2 ± 129.6 mm^3^). This indicates that the binding of dAb_PD−L1/CD24_-mPDA@CuO_2_ NRs to CD24/PD-L1 on the surface of 4T1 tumor cells resulted in the generation of •OH, T-cell activation, and secretion of IFN-γ, exhibiting a synergistic effect of CBIT and H_2_O_2_ self-supplying CDT. Finally, the potential in vivo toxicity of dAb_PD−L1/CD24_-mPDA@CuO_2_ NRs treatment was evaluated, and no significant differences in body weight among the treatment groups were observed (Fig. 7C), indicating negligible off-target side effects for all treatments.

To further confirm the ability of the dAb_PD−L1/CD24_-mPDA@CuO_2_ NRs to reactivate the T-cells via blocking PD-L1–PD1 signaling, we analyzed the secretion level of IFN-γ in the tumor environment after treatment with immunohistochemistry. As shown in Fig. 7D, higher IFN-γ secretion was observed after the dAb_PD−L1/CD24_-mPDA@CuO_2_ NRs treatment. Semiquantification showed that the tumors treated with mPDA@CuO_2_ NRs found low-density of IFN-γ^+^ CD8 T cells (398.5 ± 89.3 IFN-γ^+^ CD8 T cells/mm^2^) in the tumor area. In contrast, the tumors treated with the dAb_PD−L1/CD24_-mPDA@CuO_2_ NRs showed significantly increased density of IFN-γ^+^ CD8 T cells (1512.6 ± 178.4 IFN-γ^+^ CD8 T cells/mm^2^) in the tumor area. These results could be attributed to the high tumor accumulation of the dAb_PD−L1/CD24_-mPDA@CuO_2_ NRs via binding to CD24 and PD-L1 on the 4T1 tumor cells, which could block PD-L1–PD1 signaling to reactivate T-cells and trigger IFN-γ secretion. We have also verified that dAb_PD−L1/CD24_-mPDA@CuO_2_ NRs can reactivate macrophages by blocking CD24–Siglec-10 signaling. This was demonstrated by staining for CD68 in tumor tissues and analyzing them using immunohistochemistry post-treatment. The results, illustrated in Fig. 7E, reveal a substantial presence of CD68 + infiltrating macrophages in tumor tissues treated with dAbPD-L1/CD24-mPDA@CuO_2_ NRs compared to those treated with mPDA@CuO_2_ NRs. Taking together, dAb_PD−L1/CD24_-mPDA@CuO_2_ NRs have the capability to block both PD-L1–PD1 and CD24–Siglec-10 signaling pathways simultaneously, reactivating IFN-γ + CD8 T cells and CD68 + infiltrating macrophages in TNBC CBIT.

## Conclusions

In conclusion, this study successfully developed dAb_PD−L1/CD24_-mPDA@CuO_2_ NRs, tumor microenvironment-activated nanoreactors that effectively generated toxic •OH through H_2_O_2_ self-supply within the tumor environment. These nanoreactors exhibited the ability to reactivate T cells and infiltrating macrophages by blocking the CD24-Siglec-10 and PD-L1-PD1 signaling pathways, leading to the secretion of IFN-γ and enhanced cancer cell killing. Upon internalization by cancer cells, the dAb_PD−L1/CD24_-mPDA@CuO_2_ NRs underwent decomposition within the acidic endo/lysosomal compartments, releasing Fenton catalytic Cu^2+^ ions and H_2_O_2_. This decomposition led to the production of toxic •OH, which in turn induced lipid peroxidation and caused cancer cell death. The synergistic effect of H_2_O_2_ self-supplying CDT and CBIT demonstrated by the dAb_PD−L1/CD24_-mPDA@CuO_2_ NRs in the acidic tumor microenvironment presents a promising drug-free synergistic therapy approach for breast cancer, particularly for the treatment of triple-negative breast cancer (TNBC).

### Electronic supplementary material

Below is the link to the electronic supplementary material.

Supplementary Material 1

## Data Availability

All data generated or analyzed during this study are included in this manuscript.
