# Correction: Dual antibody-aided mesoporous nanoreactor for H_2_O_2_ self-supplying chemodynamic therapy and checkpoint blockade immunotherapy in triple-negative breast cancer

**DOI:** 10.1186/s12951-023-02233-2

**Published:** 2023-12-21

**Authors:** Ying-Tzu Chen, Ying-Xiang Luo, Shih-Hsuan Chan, Wen-Yi Chiu, Hung-Wei Yang

**Affiliations:** 1 Department of Biomedical Engineering, National Cheng Kung University, Tainan, 70101 Taiwan; 2 Department of Neurosurgery, Neuroscience Research Center, Chang Gung Memorial Hospital, Linkou, 33305 Taoyuan Taiwan; 3 Institute of Medical Science and Technology, National Sun Yat-sen University, Kaohsiung, 80424 Taiwan; 4 School of Chinese Medicine, College of Chinese Medicine, China Medical University, Taichung, 40402 Taiwan; 5 Cancer Biology and Precision Therapeutics Center, China Medical University, Taichung, 40402 Taiwan; 6 Chinese Medicine Research Center, China Medical University, Taichung, 40402 Taiwan; 7 Department of Family Medicine, Kaohsiung Armed Forces General Hospital, Kaohsiung, 80284 Taiwan; 8 Medical Device Innovation Center, National Cheng Kung University, Tainan, 70101 Taiwan

**Correction: Chen et al. Journal of Nanobiotechnology (2023) 21:385**

10.1186/s12951-023-02154-0

In this article the statement in the Funding information section was incorrectly given as ‘This research was financially backed by the National Science and Technology Council (NSTC110-2628-B-110-004, NSTC111-2628-B-006-023, NSTC112-2628-B-006-015) and National Health Research Institutes (NHRI-EX112-11226EI), Taiwan’ and should have read

‘This work was financially supported by the National Science and Technology Council (NSTC111- 2628-B-006-023 and NSTC112-2628-B-006-015), National Health Research Institutes (NHRI-EX112- 11226EI), and Kaohsiung Armed Forces General Hospital (KAFGH_D_110025), Taiwan’.

The original article [1] has been corrected.
