# Supplementary material for: Dual antibody-aided mesoporous nanoreactor for H2O2 self-supplying chemodynamic therapy and checkpoint blockade immunotherapy in triple-negative breast cancer
Source: J Nanobiotechnology. 2023 Oct 24;21:385. doi: 10.1186/s12951-023-02154-0 (PMC10594761; doi:10.1186/s12951-023-02154-0)
Supplement: Supplementary file 1 — Supplementary Material 1 [file 12951_2023_2154_MOESM1_ESM.docx]

# Supporting Information

# Dual antibody-aided mesoporous nanoreactor for H_2_O_2_ self-supplying chemodynamic therapy and checkpoint blockade immunotherapy in triple-negative breast cancer

Ying-Tzu Chen^1,2^, Ying-Xiang Luo^3^, Shih-Hsuan Chan^4,5,6^, Wen-Yi Chiu^3,7,^*, Hung-Wei Yang^1,8,^*

^1^Department of Biomedical Engineering, National Cheng Kung University, Tainan 70101, Taiwan

^2^Department of Neurosurgery, Neuroscience Research Center, Chang Gung Memorial Hospital, Linkou, Taoyuan 33305, Taiwan

^3^Institute of Medical Science and Technology, National Sun Yat-sen University, Kaohsiung 80424, Taiwan

^4^School of Chinese Medicine, College of Chinese Medicine, China Medical University, Taichung 40402, Taiwan

^5^Cancer Biology and Precision Therapeutics Center, China Medical University, Taichung 40402, Taiwan

^6^Chinese Medicine Research Center, China Medical University, Taichung 40402, Taiwan

^7^Department of Family Medicine, Kaohsiung Armed Forces General Hospital, Kaohsiung 80284, Taiwan

^8^Medical Device Innovation Center, National Cheng Kung University, Tainan 70101, Taiwan

These authors jointly supervised this work: Wen-Yi Chiu and Hung-Wei Yang. Correspondence and requests for materials should be addressed to W.Y. Chiu ([hanrogyi@gmail.com](mailto:hanrogyi@gmail.com)) and H.W. Yang ([howardyang@gs.ncku.edu.tw](mailto:howardyang@gs.ncku.edu.tw))


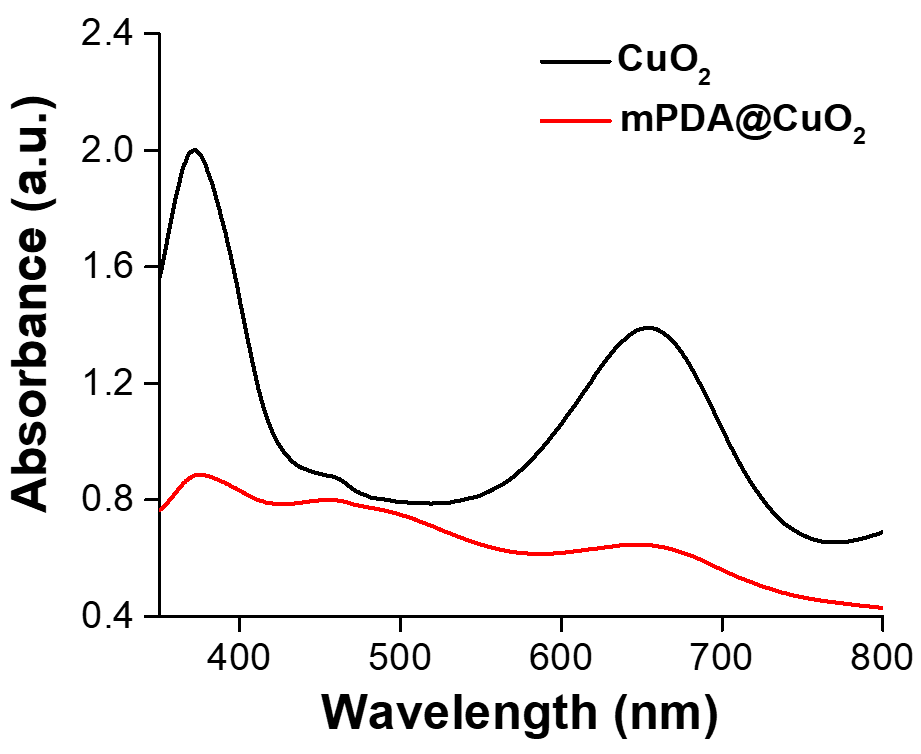


**Fig. S1.** UV-vis spectra of TMB aqueous solution incubated with 1 mM of H_2_O_2_ in the presence of CuO_2_ NRs and mPDA@CuO_2_ NRs (100 μg/mL) pretreated in an acidic solution for 30 min.

**Fig. S2.** The diameter of 4T1 tumor spheroid after treated with mPDA NPs, mPDA@CuO_2_ NRs, and dAb_PD-L1/CD24_-mPDA@CuO_2_ NRs. The values are expressed as means ± SD (n = 3). Asterisks indicate a significant difference (Student's t-test, **p* ≤ 0.05).


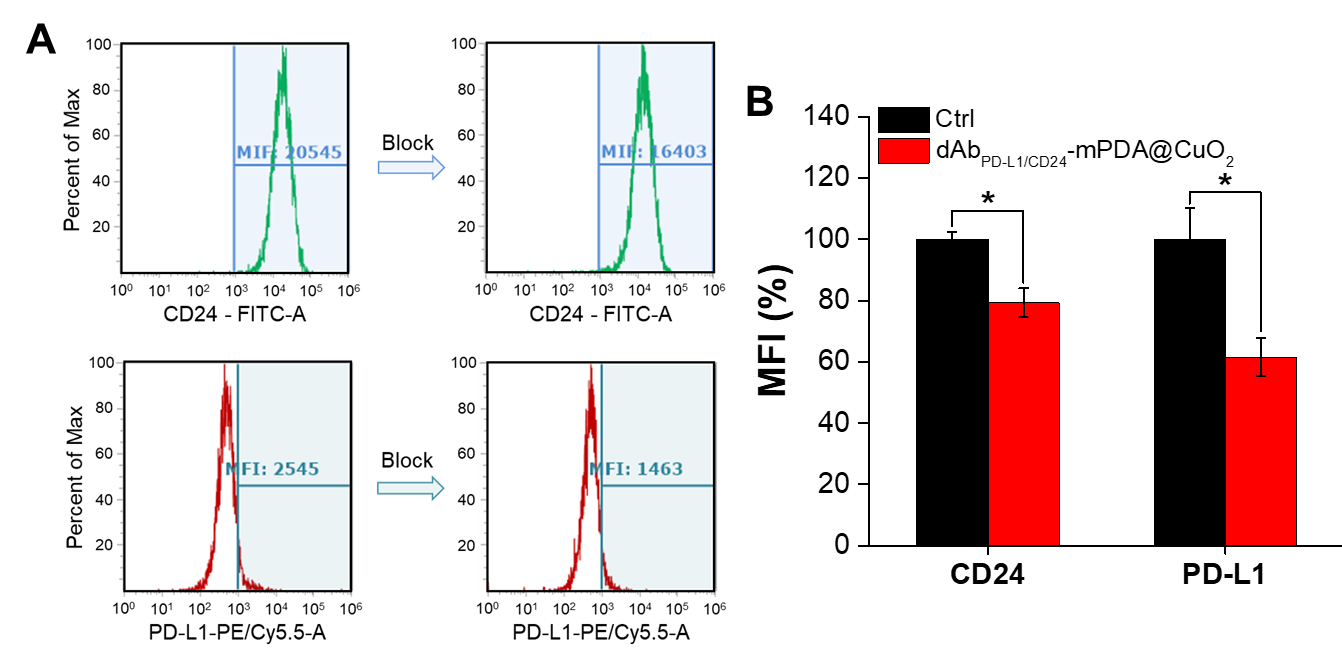


**Fig. S3.** (A) The expression of PD-L1 and CD24 proteins on the cell membrane of 4T1 cells has been analyzed through flow cytometry, both before and after treatment with dAb_PD-L1/CD24_-mPDA@CuO_2_ NRs. (B) The data are represented as the percentage of mean fluorescent intensity, calculated from the results obtained in (A). The values are expressed as means ± SD (n = 3). Asterisks indicate a significant difference (Student's t-test, **p* ≤ 0.05).
